# YAP as oncotarget in uveal melanoma

**DOI:** 10.18632/oncoscience.57

**Published:** 2014-06-22

**Authors:** Fa-Xing Yu, Kang Zhang, Kun-Liang Guan

**Affiliations:** Department of Pharmacology and Moores Cancer Center, University of California at San Diego, La Jolla, California

Uveal melanoma (UM) is the most common intraocular malignancy in adults. Currently, the affected eye is either removed by surgery (enucleation) or treated with radiation therapy. However, there is no effective treatment for metastatic UM, which frequently spreads to the liver via a haematogenous route.

The majority (~80%) of UM cases are driven by mutually exclusive mutations in *GNAQ* or *GNA11*, the genes encoding two homologous alpha subunits (Gα_q_ or Gα_11_, hereafter referred to as Gq/11) of the heterotrimeric G-protein [1, 2]. These mutations are concentrated on amino acid residue glutamine 209 (Q209) with a much lower mutation frequency on arginine residue 183 (R183), turning Gq/11 constitutively active, thus oncogenic [1, 2]. These genetic studies provide an attractive possibility of a targeted molecular therapy for UM. Unfortunately, no specific inhibitor targeting mutant Gq/11 is available. However, mutant Gq/11 may activate downstream signaling events to promote UM, therefore defining the key molecular mechanisms involved in mutant Gq/11-induced carcinogenesis may provide novel drug targets for UM intervention.

The Hippo pathway plays a critical role in regulating tissue growth and organ size, and its dysregulation leads to neoplastic growth and cancer [3]. Yap, the major effector of the Hippo pathway, is an oncoprotein, and its activation is frequently observed in multiple human cancers. Yap activity is repressed by upstream Hippo pathway components, such as Large tumor suppressor kinases Lats1/2 [3]. When Yap is phosphorylated by Lats1/2, it is sequestered in the cytoplasm and inactive. Conversely, when Yap is dephosphorylated, it accumulates in the nucleus where it interacts with cognate transcription factors to induce gene expression and promote cell proliferation [3]. We have previously demonstrated that the Hippo pathway is modulated by G-protein-coupled receptor (GPCR) signaling, and active Gq/11 can activate YAP by inhibiting Lats1/2 kinase activity [4]. These findings suggest that the Hippo pathway may contributes to development of tumors containing mutant Gq/11, such as UM.

In two recent reports in Cancer Cell, we and Gutkind's team have independently shown that YAP mediates the oncogenic activity of mutant Gq/11 in UM [5, 6]. Accumulation of dephosphorylated (active) Yap in the nucleus is observed in multiple cell lines derived from UMs with Gq/11 mutations, and Yap nuclear localization correlates with Gq/11 mutations in UM patient samples [5, 6]. In a transgenic mouse model, expression of mutant Gq, driven by lineage-specific promoters, results in cutaneous melanoma or skin carcinoma, and Yap is also nuclear in these tumors [5]. When mutant Gq is knocked down in UM cell lines, Yap phosphorylation is increased and Yap nuclear localization is decreased. These results indicate that Yap activity is elevated in mutant Gq/11-induced primary human UMs and mouse tumors. Moreover, when Yap is knocked down, tumor growth of Gq mutated UM cells in nude mice is significantly blocked, indicating an essential role of Yap in mediating the oncogenic effect of mutant Gq/11 in UM.

An exciting observation described in the two studies is that Verteporfin, a known Yap inhibitor [7], suppresses UM tumor growth in mouse models. When Verteporfin is given to mice after UM cells have been grafted subcutaneously [5] or orthotopically into the eye [6], tumor growth of UM cells harboring the Gq mutation is significantly blocked. Verteporfin is an FDA approved drug for photodynamic therapy (PDT) used to treat abnormal blood vessel formation in the eye, thus these two studies provide proof-of-concept for UM treatment by targeting YAP and also suggest the possibility of repositioning Verteporfin for UM treatment. Verteporfin may have two different mechanisms in UM treatment: inhibiting angiogenesis, which requires photosensitization, or blocking Yap activity, which does not require photosensitization. As mentioned in Gutkind's report, PDT using Verteporfin as a photosensitizer has already been used to treat limited numbers of UM patients with encouraging outcomes. However, drug administration may be difficult because Verteporfin's solubility is very low. Systematic delivery by intraperitoneal injection was not very successful in our hands, therefore we have designed nanoparticles to package Verteporfin for delivery locally to mouse eyes. Exploring new Yap inhibitors with better pharmacological properties than Verteporfin would be very beneficial for future UM treatment by targeting Yap.

**Figure 1:**
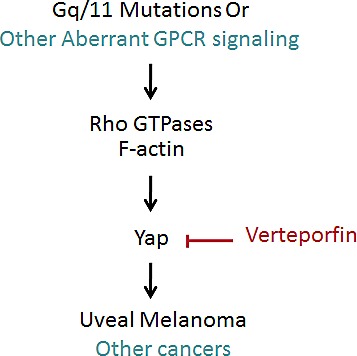
Mutant Gq/11 signal through YAP to promote uveal melanoma

Activating Gq/11 mutations and dysregulated GPCR signaling are also present in other types of cancer [8]. Especially, mutations in RhoA, a strong upstream activator of YAP, have been recently found in human cancers. Moreover, mutations in Hippo pathway components, such as Nf2, are also observed in schwannoma, meningioma, and mesothelioma, in which YAP activation may play a major role. Therefore, targeting YAP may prove to be a fruitful approach for treatment of multiple human cancers with aberrant YAP activity.
